# Chloroplast Genes Are Involved in The Male-Sterility of K-Type CMS in Wheat

**DOI:** 10.3390/genes13020310

**Published:** 2022-02-07

**Authors:** Yucui Han, Yujie Gao, Yun Li, Xiaoguang Zhai, Hao Zhou, Qin Ding, Lingjian Ma

**Affiliations:** 1 College of Agronomy and Biotechnology, Hebei Normal University of Science and Technology, Qinhuangdao 066000, China; yucuihan84@163.com (Y.H.); liyun0118@126.com (Y.L.); 2 College of Agronomy, Northwest A&F University, Xianyang 712100, China; gaoyujie8000@163.com (Y.G.); zhaixiaoguangdkl@163.com (X.Z.); nongdahaozhou@163.com (H.Z.); 3 College of Horticulture, Northwest A&F University, Xianyang 712100, China

**Keywords:** wheat, chloroplast genome, male sterility, SSR, gene-silencing

## Abstract

The utilization of crop heterosis can greatly improve crop yield. The sterile line is vital for the heterosis utilization of wheat (*Triticum aestivum* L.). The chloroplast genomes of two sterile lines and one maintainer were sequenced using second-generation high-throughput technology and assembled. The nonsynonymous mutated genes among the three varieties were identified, the expressed difference was further analyzed by qPCR, and finally, the function of the differentially expressed genes was analyzed by the barley stripe mosaic virus-induced gene silencing (BSMV-VIGS) method. A total of 16 genes containing 31 nonsynonymous mutations between K519A and 519B were identified. There were no base mutations in the protein-encoding genes between K519A and YS3038. The chloroplast genomes of 519B and K519A were closely related to the *Triticum* genus and *Aegilops* genus, respectively. The gene expression levels of the six selected genes with nonsynonymous mutation sites for K519A compared to 519B were mostly downregulated at the binucleate and trinucleate stages of pollen development. The seed setting rates of *atpB*-silenced or *ndhH*-silenced 519B plants by BSMV-VIGS method were significantly reduced. It can be concluded that *atpB* and the *ndhH* are likely to be involved in the reproductive transformation of 519B.

## 1. Introduction

Wheat (*T. aestivum* L.) is the world’s most widely grown food crop, feeding nearly half of the world’s population [1,2]. It is also an important raw material for industry. For example, wheat gluten can be used as a natural binder in the manufacture of paper [3]. Additionally, dry gluten extracted from wheat can be used as an additive for bread processing [4]. Improving yield per unit area and stress resistance of wheat are very effective strategies to alleviate the food problem. The utilization of heterosis is an effective way to improve wheat yield and quality, and it plays an essential role in the breeding of crops [5,6].

Wheat is a monoecious, self-pollinated crop. Therefore, male sterile lines are essential for the utilizing of heterosis. Male sterility in plants refers to the development of gynoecium as normal during the growth process, but fertilization and seed setting after receiving pollen are abnormal, which are caused by the abnormal development of stamens. The abnormal development of stamens means that the pollens are abortive or anther tissue structure is abnormal, which eventually leads to the decline or loss of pollen vitality. The causes of male sterility in plants are various. According to the origin of male sterile genes, male sterile lines can be divided into three types: genic male sterility (GMS), cytoplasmic male sterility (CMS), and nucleus-cytoplasmic male sterility. At first, Kihara [7] transferred the nucleus of common wheat into *Aegilops caudata* and cultivated the first nucleus-cytoplasmic male sterile line in 1951. Wilson and Ross [8] introduced the common wheat nucleus into the *Triticum timopheevi*. Finally, they successfully selected and obtained T-type CMS wheat. After this experiment, the maintainer and restorer lines were also selected, these studies were important for the utilization of wheat heterosis [9]. After years of unremitting efforts by scientists, there are many different cytoplasmic sterile lines that have been cultivated in China [10]. The K-type wheat sterile line has the *Aegilops* *kotschyi* cytoplasm, S-type has *Aegilops crassa* cytoplasm, Q-type has wild oat cytoplasm, and V-type has *Aegilops variabilis* cytoplasm, and they all have the nucleus of common wheat. In addition, according to the response environments, male sterile lines were divided into thermo-sensitive male sterile lines, photosensitive male sterile lines, and photothermosensitive male sterile lines.

The K-type 1B/1R CMS line is a heterozygote in the cytoplasm of *Ae. kotschyi* and 1B/1R nucleus of *T. aestivum* [11]. On this basis, the K-type non-1B/1R CMS line was cultivated. The sterility of the K-type non-1B/1R CMS line was complete and stable, as well as the recovery sources and agronomic traits, thus it has great application value. There have been some researches about K-CMS in physiology [12], cytology [13], protein expression [14], and gene function analysis [15]. For instance, Yang et al. [15] cloned and analyzed the functions of MADS-box, *TaAG-A*, and *TaAG-B* genes from a wheat K-CMS line. They found that the expression levels of *TaAG-A* and *TaAG-B* were higher in spikes. In the maintainer line, both genes showed downregulation during the uninucleate to trinucleate stage. Then the *TaAG-A* and *TaAG-B* were silenced in fertile wheat lines using the VIGS method, resulting in green and yellow striped leaves, emaciated spikes, and decreased seed set rates. Zhang et al. [14] found a total of 41 protein spots differentially expressed through proteomic analysis of pollen in K-CMS lines and maintainers, of which, seven identified proteins were downregulated in CMS lines.

In general, the genetic systems of higher plants mainly include the nuclear genome and the cytoplasmic genome. The cytoplasmic genome mainly includes the mitochondrial and chloroplast genomes. Both mitochondria and chloroplast inheritance belong to the semiautonomous genetic system, which is capable of autonomous DNA replication, transcription, and translation [16]. Besides, many physiological metabolisms of plant cells depend on the combined action of the nucleus, mitochondria, and chloroplast [17,18]. In higher plants, a phenomenon is that the genes of chloroplast and nucleus can be transferred to the mitochondria. Additionally, the chloroplast genes that are transferred to mitochondria often evolve into nonfunctional pseudogenes [19]. However, the genes in the nucleus and mitochondria typically are not transferred to the chloroplast [20,21]. Besides, this process is bidirectional between the mitochondrial and nuclear genomes [22,23]. We speculate that the combination of different types of cytoplasm and nucleus may destroy the intrinsic balance between chloroplast, nucleus, and mitochondria, which may lead to the formation of cytoplasmic male sterility.

Many studies have revealed that mitochondrial genes are involved in plant male sterility, including *atp* [24,25], *cox* [24], *rps* [24], ORF [26,27], and ORF interacted with genes [28]. The common modes of male sterility were rearrangements of mitochondrial genes and chimeric gene fusion [29,30,31]. In addition, the chimeric CMS genes are mostly transcribed together with the upstream or downstream genes, such as *ORF79* is co-transcribed with the upstream *atp6* gene in rice [32]. *Nad2* is co-transcribed with *orfB* in stem mustard (*Brassica juncea*) [33]. Cytoplasmic male sterility consists of three main pathways: Mitochondrial genes can directly cause male sterility by energy deficiency [34]; male sterility can be caused by the interaction of cytoplasmic and nucleus genes, due to the retrograde signaling from the mitochondrion interferes with nuclear gene expression [35] and there are also some mitochondrial genes that cause male sterility through differences in expression [24].

Chloroplasts are important organelles for photosynthesis in higher plants and are important organelles to independent genetic information in cells. The chloroplast genome, also known as chloroplast DNA, refers to the sum of the genetic information contained in the chloroplast [36]. Most chloroplast genomes are 120–250 kb in size and chloroplast genomes typically are depicted as closed double-stranded circular rings [37]. They are typically four-segment structures consisting of two inverted repeats (IRs), a short single copy region (SSC), and a long single copy region (LSC). The sequence of the IRs’ regions is the same but in the opposite direction. The structures of the chloroplast genomes are relatively conservative and have fewer changes during the evolution of species. Therefore, the chloroplast genome can provide reliable and accurate information for biodiversity research and provide a lot of basic information for comparative evolution research [38,39].

Since the chloroplast genes can be transferred to mitochondria as mentioned above, and since many mitochondrial genes are involved in the process of male sterility in plants, chloroplast genes might affect male sterility by changing the gene function of mitochondria. Besides, some special structural compositions in chloroplasts were also considered related to the fertility of plants [40]. Li et al. [41] used SDS-PAGE and two-dimensional electrophoresis to compare the differences of peptides on chloroplast thylakoid membranes between CMS lines and their maintainers in beet, corn, and sorghum. There were significant differences in the size, number, and distribution of peptide plaques between the two lines, proving that the composition of chloroplast thylakoid polypeptides might affect cytoplasmic male sterility. Li et al. [42] studied the chloroplast ultrastructure of the CMS line and its maintainer in rape, the number of stacks of grana in the thylakoid of the sterile line was significantly reduced. Additionally, the lamellar structure between grana became thinner or even fractured so that the lamellar system was disordered. Chen et al. [43] found that after the digestion of chloroplast genome DNA of sorghum male sterile line and maintainer line with *HindIII* endonuclease, the male sterile line produced a 3.7 kb specific fragment, with a 165 bp lack compared to the maintainer line (3.8 kb). The deletion fragment was from the *rpoC2* gene, which encodes the β subunit of the RNA polymerase. The amino acids of the deletion fragment will participate in the formation of an α helix. It was found that the identified 1501 differentially expressed transcripts in leaves and anthers at different developmental stages were most of these DETs being localized in plastid and mitochondrion of male sterile line in *Brassica napus* L. induced by the chemical hybridization agent compared to the wild *Brassica napus* L. [44].

K519A is a non-1B/1R K-CMS line, and 519B was the maintainer line of K519A with the same nuclear genotype. Their nuclear genomes are derived from the same paternal species, but their cytoplasm genomes are from different maternal species. Thus, the differences in fertility come from the differences between cytoplasmic genomes. YS3038 is also a non-1B/1R K-CMS line. Additionally, the nucleus of YS3038 is basically the same as that of K519A. However, the sterility of YS3038 is thermo-sensitive. To understand the sterile mechanism of K519A and YS3038, the chloroplast genomes of K519A, YS3038, and 519B were sequenced, assembled, annotated, and compared in this study. Then the nonsynonymous mutant sites of coding genes were verified. Besides, possible SSR, possible long repeat sequences, and phylogeny were analyzed. This study can provide an important reference value for the sterile mechanism studies of K519A and YS3038.

## 2. Materials and Methods

### 2.1. Material Planting

The K-type male sterile line K519A (non-1B/1R *T. aestivum Ae. kotschyi* cytoplasm), YS-type thermo-sensitive male sterile line YS3038 (non-1B/1R *T. aestivum* with *Ae. kotschyi* cytoplasm), and the same nuclear genotype maintainer line 519B (*T. aestivum*) of K519A were used in this study. They are provided by our laboratory (Northwest A&F University, Yangling, Shaanxi, China).

**Figure 1:**
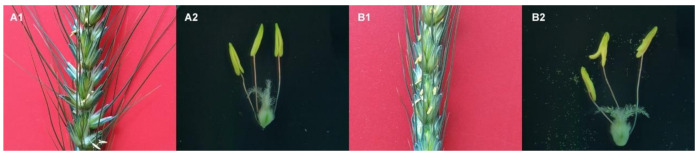
The structure observation of the ear and anther of male sterile line and 519B. (**A1**,**A2**) male-sterile line. (**B1**,**B2**) 519B.

After the pollens matured, the opening angle of the glumes of the male sterile line become larger than 519B, the anthers stretch out from the glume (Figure 1(A1,B1)). Additionally, the anther is wizened, short, and crooked, and the pollen cannot spread out from the anther (Figure 1(A2)). After the pollens have matured, the anthers of 519B are plump and cracked, and the amount of fertile pollen spreads out from the anthers (Figure 1(B2)).

The test materials were planted in the experimental field of the Northwest A&F University (108°4′ E, 34°16′ N) on 3 October 2018. The two materials were planted in 4 rows (25 cm between rows), respectively, with 15 seeds in 1 m. Planting was done with general field management measures. Young leaves were stored in a refrigerator at −80 °C in March 2019.

### 2.2. CTAB Extraction of DNA and Chloroplast Genome Sequencing

Genomic DNA from young leaves of K519A, YS3038, and 519B wheat was extracted by means of the CTAB method. The whole-genome DNA sequences were obtained using second-generation high-throughput technology, with the Illumina HiSeq XTM Ten sequencing platform and paired-end 150 bp sequencing strategy. Firstly, the original sequencing data for each sample was obtained, then low-quality reads and connector sequences were removed. Additionally, the clean data was used for further genome assembly of the chloroplast.

### 2.3. Genome Assembly and Annotation

The chloroplast assembly was carried out based on the assembly method of Hahn et al. [45]. The assembly steps are as follows: Firstly, the clean data is spliced using SPAdes software [46], setting default parameters except that the cut-off parameter is not selected. The clean data are spliced into scaffolds. Using the published wheat chloroplast DNA sequences (Chinese Spring, CS, *T. aestivum*) and protein-coding gene sequences as a reference (KJ614396.1). Then the sequences are aligned using Blastn and Exonerate, respectively. The e value threshold of DNA alignment is 1 × 10^−10^, and the protein similarity threshold is 70%, respectively. Scaffold with gene matching was selected and the coverage was ranked to remove fragments that were clearly not the target fragments, such as most of them were 1000X, but a few were 10X, and then the low-coverage fragments (10X) were removed. PRICE and MITObim was used to splice the collected fragmented sequences to extend the scaffolds. The number of iterations generally was 50. The original sequencing reads were aligned to scaffolds using bowtie2, then the matched paired reads were picked out and spliced by SPAdes. The path was studied to see if a distinct circle diagram could be formed. If so, the circle genome was extracted, otherwise, steps 3–5 above were repeated until a circular genome was formed.

The organelle genome annotation is mainly divided into three parts: protein-coding gene annotation, RNA annotation, and structural annotation. Three methods are commonly used for protein-coding gene prediction. The DNA sequences or protein-coding sequences directly align with NCBI to confirm the genes. Submitting sequences to the online service annotation tools, DOGMA (http://dogma.ccbb.utexas.edu/, accessed on 7 April 2020) and CpGAVAS [47], predicting the ORFs, and then annotating genes in the nr database. In combination with the three methods, the most accurate annotation results are chosen.

Chloroplast tRNA annotations were performed using the tRNAscan-SE online site (http://lowelab.ucsc.edu/tRNAscan-SE/, accessed on 15 May 2020). The rRNA annotations were performed using the RNAmmer 1.2 Server (http://www.cbs.dtu.dk/services/RNAmmer/, accessed on 28 May 2020), in combination with homologous alignments, and boundary correcting. After the sequence annotation was completed, the structure note was edited by Sequin to generate a submission file that could be submitted to the GenBank database. The file was submitted to OGDRAW (https://chlorobox.mpimp-golm.mpg.de/OGDraw.html, accessed on 17 June 2020) to draw the annotated map.

### 2.4. Codon Preference Analysis of Chloroplast Gene

A codon is a link between genetic information from DNA and protein, and every three adjacent codons encode an amino acid. In all organisms in nature, there are only 20 amino acids in total, however, each amino acid corresponds to at least one or up to six codons. Synonymous codons mean that different codons can encode the same amino acid [48]. The analysis of codon preference was performed using the CHIPS program in the EMBOSS software package [49].

### 2.5. Simple Sequence Repeat (SSR) and Long Repeat Sequence Analysis

The Microsatellite identification tool (MiSa) [50] was used for finding simple sequence repeats (SSR) in the genome. We used this software to detect SSR with eight times as a minimal repeat number of mononucleotides, five times for dinucleotides, and at least three times for three or more bases.

The polymorphism SSR markers at the same position in the same gene of K519A and 519B were selected. Primer Premier 6.0 software was used to design SSR primers to verify the polymorphism between K519A and 519B.

Using REPuter online software [51] to find long repeat sequences, all options: forward, reverse, complement, and palindromic were selected for “Match Direction”. The parameter “Hamming distance” was set to 3, “maximum computed repeat” to 50, and “minimal repeat size” to 15.

### 2.6. Gene Alignment Analysis and Phylogenetic Analysis

We used MEGA4.0 software [52] to align the homology of protein-coding genes, then we selected all nonsynonymous mutant sites to verify the accuracy of second-generation sequencing results. According to the company’s sequencing sequence, Primer Premier 6.0 software was used to design primers of nonsynonymous mutant sites, then PCR products were sent to the company for first-generation sequencing (FGS).

The genome-wide alignment of chloroplast genomes K519A, 519B, YS3038, and another 100 chloroplasts of near-source species were performed using HomBlocks (https://github.com/fenghen360/HomBlocks, accessed on 22 July 2021) software with the trimAl trim method. The NJ method was used to build the evolutionary tree with bootstrap value 1000.

### 2.7. Quantitative Real-Time PCR (qPCR) Analysis

Premier 6.0 software was used for gene-specific primers. RevertAid First Strand cDNA Synthesis Kit (Thermo Scientifc, Wilmington, DE, USA) was used for cDNA synthesis with random primers according to instructions. TB GreenTM Premix Ex TaqTM II (Tli RNaseH Plus) Kit (Takara Biological Engineering, Tokyo, Japan) and Applied Biosystems 7300 Real-Time PCR System (Life Technologies, Carlsbad, CA, USA) were used for the qPCR analysis with three biological replicates and three technical replicates. The wheat Actin gene with AB181991.1 (Gene Bank) was used for normalization. The 2^–ΔΔCT^ method was used to calculate relative expression levels of target genes.

### 2.8. Functional Verification of Candidate Genes via the BSMV-VIGS Method

#### 2.8.1. Construction of γ-Gene Vector

The approximate 200 bp gene fragment with the *PacI* (TTAATTAA) and *NotI* (GCGGCCGC) enzyme locus were used for gene-silenced. Then the vector of γ-gene was obtained.

#### 2.8.2. Linearization and in Vitro Transcription of γ-Gene Vector

*MluI* was used for the linearization of α and γ plasmids; *SpeI* was used for the linearization of β plasmid; and *BssHII* was used for the linearization of γ-PDS and γ-gene plasmids. Then, in vitro transcription was performed using Ribo m^7^G Cap Analog (Promega), RiboMAX Large Scale RNA Production System-T7 (Promega), and Ribolock RNase Inhibitor (Thermo).

#### 2.8.3. Creation of Transfection Mixture and Virus Infection of Seedlings

The negative control plants used in vitro transcript mixture of α, β, and γ, while the positive control plants used in vitro transcript mixture of α, β, and γ-PDS, and the treatment plants used an in vitro transcript mixture of α, β, and γ-gene. The 247 μL mixture could be used to infect five individuals including 7 μL in vitro transcription product for each vector, 40 μL sterilized 1% DEPC water, and 200 μL GK-Pbufer.

The penultimate leaves and the fully unfolded flag leaves were smeared with above-mentioned mixture. The plants were then placed in a 24–26 °C incubator for dark cultivation for 24 h.

#### 2.8.4. Detection of Silencing Efficiency and Phenotypes

After virus infection, 4–5 spikelets per ear were collected at the binucleate stage for RNA extraction and qPCR analysis. When ears grew to the mature stage, the seed setting rate was calculated as follows: Seed setting rate per ear = number of grains per ear / (effective spikelet number × 2) × 100%.

## 3. Results

### 3.1. Genome Sequencing and Chloroplast Assembly

Genomic sequencing was performed on K519A, 519B, and YS3038, and the number of paired-end reads of 150 bp was greater than or equal to 14,716,209 for each sample, and the number of reads was greater than or equal to 14,705,908 (4,406,664,764 clean data) and Q30 was greater than 92.35% for each sample after quality control. The length of the chloroplast genome was about 136 kb, indicating a sequencing coverage of 40×.

### 3.2. Genome Content and Characteristics

**Figure 2:**
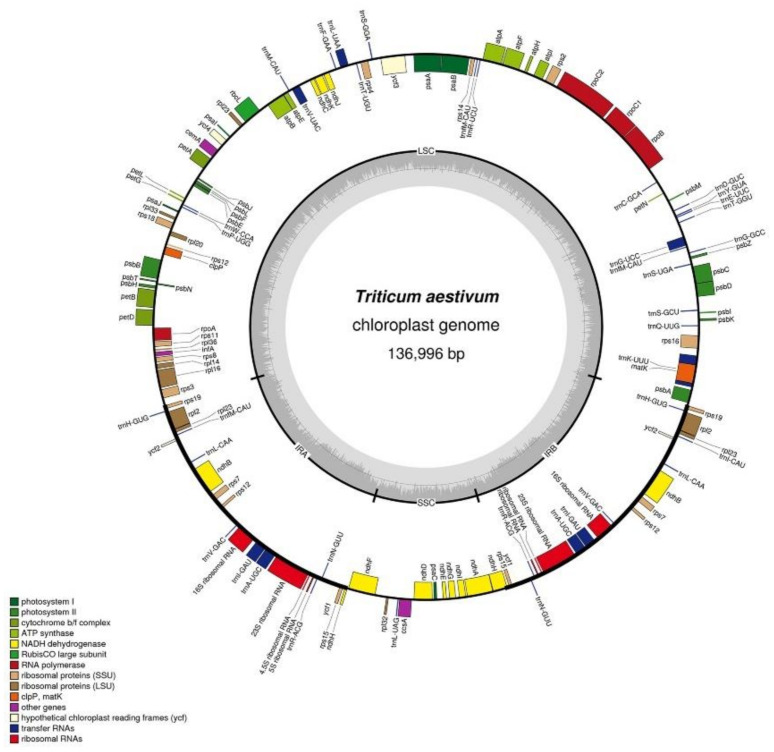
Annotated map of the chloroplast genome structure in K519A. The transcripts of inside genes are in a clockwise direction, while the transcripts of outside genes are in the opposite direction. Different functional genes are identified with different colors. The gray histogram shows genomic GC content, and the middle gray line is the 50% threshold line.

**Figure 3:**
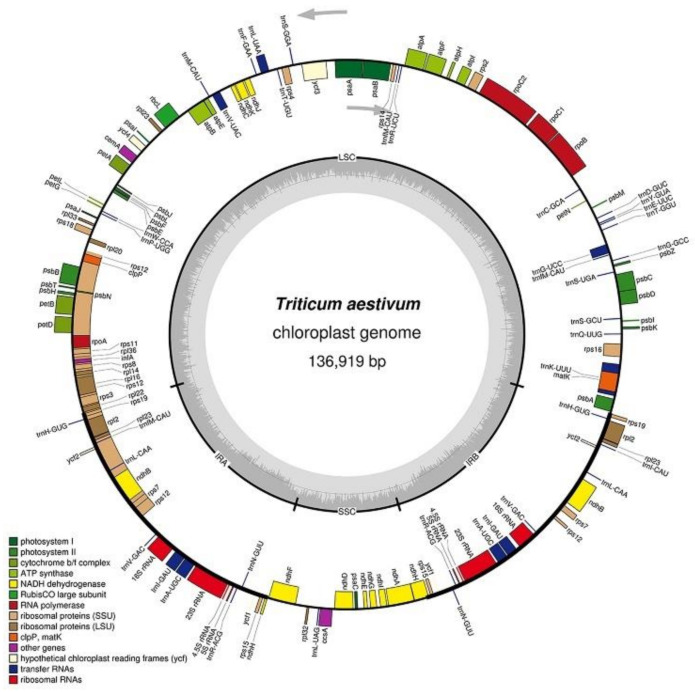
Annotated map of the chloroplast genome structure of YS3038. The transcripts of inside genes are in a clockwise direction, while the transcripts of outside genes are in the opposite direction. Different functional genes are identified with different colors. The gray histogram shows genomic GC content, and the middle gray line is the 50% threshold line.

**Figure 4:**
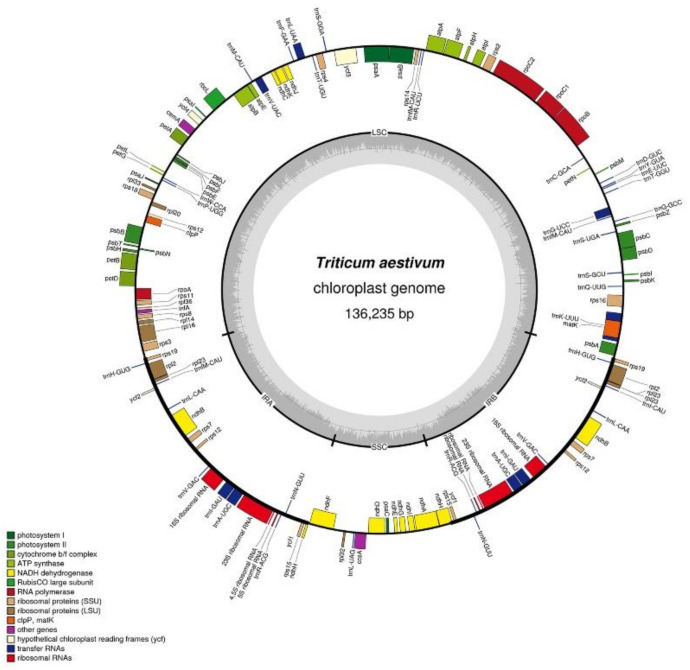
Annotated map of the chloroplast genome structure in 519B. The transcripts of inside genes are in a clockwise direction, while the transcripts of outside genes are in the opposite direction. Different functional genes are identified with different colors. The gray histogram shows genomic GC content, and the middle gray line is the 50% threshold line.

Like the chloroplast genomes of other higher plants, the two chloroplast genomes in this experiment contained two inverted repeats, IRA and IRB, which divided the entire genome into four parts, and the remaining regions were the large single-copy region (LSC) and the small single-copy region (SSC). The total length of the chloroplast genome of K519A was 136,996 bp, and the lengths of the four parts were 81,136 bp (LSC), 12,776 bp (SSC), and 21,542 bp (IRA and IRB) (Figure 2). The total length of the YS3038 was 136,919 and the lengths of the four parts were 81,059 bp (LSC), 12,776 bp (SSC), and 21,542 bp (IRA and IRB) (Figure 3). The total length of 519B was 136,235 bp, and the lengths of the four parts were 80,335 bp (LSC), 12,796 bp (SSC), and 21,552 bp (IRA and IRB) (Figure 4). The GC contents of genomes were 38.27% (K519A), 38.28% (YS3038), and 38.28% (519B), respectively. The structures of the chloroplast genomes of the two male sterile lines and 519B were different, such as the expansion and contraction of the boundaries of the four parts, and the sequence insertion of some regions.

All chloroplast genomes include 77 protein-coding genes, 4 rRNA genes, and 30 tRNA genes. Based on the functions, chloroplast genes and RNA were mainly divided into three categories: photosynthesis (45), self-replication (58), unknown function (4), and others (4) (Table 1). Of these, there were two copies of the protein-coding genes *ndhB*, *ndhH*, *rps7*, *rps15*, *rps19*, *ycf1*, *ycf2*, and *rpl2*. Additionally, there were three copies of *rpl23*. In the tRNA genes, *trnA-UGC*, *trnH-GUG*, *trnI-GAU*, *trnN-GUU*, *trnR-ACG*, and *trnV-GAC* contained two copies. Additionally, there were three copies of the *trnfM-CAU*. Besides, the *trnL-CAA* gene had two copies only in YS3038. The *trnA-UGC*, *trnG-UCC*, *trnI-GAU*, *trnK-UUU*, *trnL-UAA*, *trnV-UAC*, *atpF*, *ndhA*, *ndhB, petB*, *petD*, *rpl2*, *rpl16*, and *rps16* contained an intron, and *ycf3* contained two introns. *Rps12* is a trans-splicing gene.

The regions of exons and introns for each gene were predicted, and the codon preferences were analyzed based on the sequence of exons. The codon preferences of K519A and 519B were different. Additionally, the codon preferences of genes in K519 and 519B are shown in Table 2 and Table 3, respectively. Among them, both Met and Trp were encoded by only one codon. Ile and stop codons were encoded by three codons. Ala, Gly, Pro, Thr, and Val were encoded by four codons. Arg, Leu, and Ser were encoded by six codons.

### 3.3. Discovery of Possible SSRs in K519A and 519B

The SSR markers of chloroplasts have been applied for studies of population genetics, evolution processes, and phylogeny [53,54]. In this study, we found 186 possible SSR repeat motifs in K519A (Table 4) and 188 in 519B (Table 5). In SSRs, the mononucleotide repeats were the most abundant compared to polynucleotide repeats, and the number of A/T repeat motifs was significantly higher than the number of C/G, which was consistent with the results of six legume plastid genomes [55]. In K519A, the proportion of single nucleotide repeat motif was 68.28% and was 67.02% in 519B. The SSR number composed of dinucleotide was 8, of which 6 were AT/TA repeat motifs and 2 were TC in K519A. The SSR number composed of dinucleotide was 9, of which 7 were AT/TA repeat motifs and 2 were TC in 519B. Among the trinucleotides, AAC/TTC most frequently appeared. Additionally, the SSR number of the remaining repeat motif types appeared only once.

### 3.4. Discovery of Possible Long Repeat Sequences in K519A and 519B

Besides SSR analysis, we also analyzed possible long repeat sequences of the chloroplast genome. Long repeat sequences are special DNA sequences that occur repeatedly in the genome and usually occupy a large proportion in the genome. In general, the proportion of repeat sequences is positively related to the size of the genome. For example, the *Arabidopsis thaliana* genome is 120 Mb, and the repeat sequences account for 10%. Most repeat sequences accounting for 81% of the wheat genomes (17,000 Mb) exist in the intergenic region, and a small number of repetitive sequences exist in the gene coding region [56]. Repetitive fragments have important molecular significance for plant evolution research [57]. In K519A, a total of 50 possible long repeats were found, and the sizes were 20–286 bp. Of the 50 possible repeats, the positive repeat, palindrome repeats, and inverted replicates were 37, 11, and 2, respectively. The longest repeat was the forward repeat with 286 bp in length. In 519B, a total of 52 possible long repeats were found, the sizes of which were also 20–286 bp, which was consistent with K519A, of which the positive repeat, palindrome repeat, and inverted repeat were 39, 10, and 3, respectively. Most of the long repeats between the two lines are consistent, only a few are inconsistent (Table 6).

### 3.5. Alignment for Protein-Coding Genes between CS, 519B, K519A, and YS3038

Firstly, the CS was used as a reference sequence to compare the protein-coding genes of 519B, 519A, and YS3038. Compared with CS, 519B has 4 genes with nonsynonymous mutations, 15 genes with synonymous mutations, a total of 28 base mutations, 4 amino acid mutations, 7 deletions, and 2 insertions. There were no base mutations in the protein-encoding genes between K519A and YS3038. Compared with CS, both K519A and 519B have 15 nonsynonymous mutated genes and 27 synonymous mutated genes, including 113 base mutations, 30 amino acid mutations, 8 deletions, and 3 insertions (Table 7). It was concluded that compared with the protein-coding gene of CS, 519B has the least synonymous mutation and nonsynonymous mutation genes, base mutation and amino acid mutation sites, insertions, and deletions, which also indicates that the cytoplasm of 519B belongs to the common wheat cytoplasm.

A comparative analysis of K519A and 519B revealed that 104 SNPs were identified in the protein-coding sequence in 42 genes (Table 7). Among them, 73 were synonymous mutations, 31 were nonsynonymous mutations from 16 genes (*atpB*, *atpI*, *ccsA*, *matK*, ndhA, *ndhF*, *ndhH*, *ndhK*, *psbB*, *psbH*, *rbcL*, *rpl14*, *rpl32*, *rpoB*, *rpoC2*, and *rps16*), and the proportion of nonsynonymous mutations was 29.8% of the total mutations. Of non-synonymous mutation genes, the NADH dehydrogenase genes are the most, and four genes occur with mutations. The protein-coding gene with the largest difference between K519A and 519B sequences was the *rpoC2* gene, and there were 13 SNPs with 5 amino acid differences.

### 3.6. The Verification of Nonsynonymous Mutant Sites between K519A and 519B by First-Generation Sequencing

**Figure 5:**
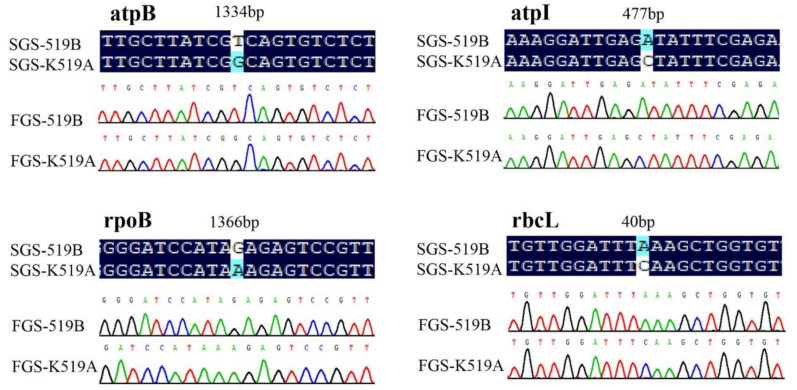
Verification of gene sequences of nonsynonymous mutated sites. SGS-K519A—second-generation sequencing sequence of K519A. SGS-519B—second-generation sequencing sequence of 519B. FGS-K519A—first-generation sequencing sequence of K519A. FGS-519B—first-generation sequencing sequence of 519B.

To verify the accuracy of second-generation sequencing results, 31 nonsynonymous mutant sites from 16 genes, selected based on the results of the above alignment analysis, were identified via FGS technology. The corresponding primers were listed in Table 8, which were prefixed with FGS. It was concluded that the amino acids of the 16 genes were all consistent under the two methods. Some comparison results were listed in Figure 5.

### 3.7. Phylogenetic Analysis of K519A and 519B

**Figure 6:**
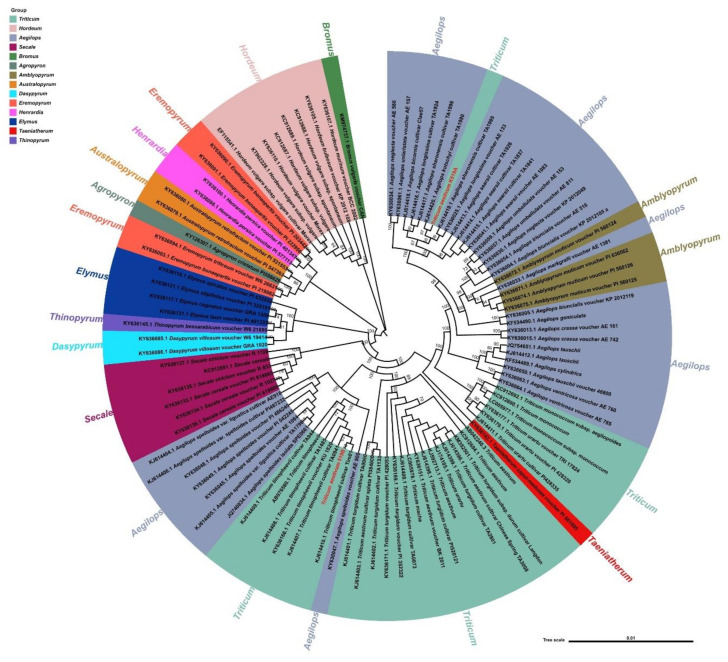
Phylogeny of chloroplast genomes of proximal species.

The phylogenetic analysis of chloroplast genomes of K519A, 519B, and another 100 species that have near relatives was performed (Figure 6). *Bromus vulgaris* was used as the outgroup. It was found that the species of the *Aegilops* genus and the *Triticum* genus were clustered in the largest number through phylogenetic analysis. Besides, four species of the *Amblyopyrum* genus were clustered into the *Aegilops* genus and a *Taeniatherum* genus cultivar was clustered into the *Triticum* genus. Then the *Secale* genus and the *Elymus* genus were clustered, but a species of the *Thinopyrum* genus and two species of the *Dasypyrum* genus were clustered in the *Elymus* genus. The relationships between the *Hordeum* genus and *Aegilops* genus were relatively distant. In the *Eremopyrum* genus, five other genus species were clustered. It was concluded that the chloroplasts of K519A and 519B were from two independent taxa, the chloroplast of 519B was closely related to the *Triticum* genus species. However, K519A was closely related to the *Ae. kotschyi* cultivar and other *Aegilops* species, which was consistent with the characteristic that K519A’s cytoplasm was derived from *Ae. kotschyi*.

### 3.8. The qPCR of Nonsynonymous Mutation Genes

**Figure 7:**
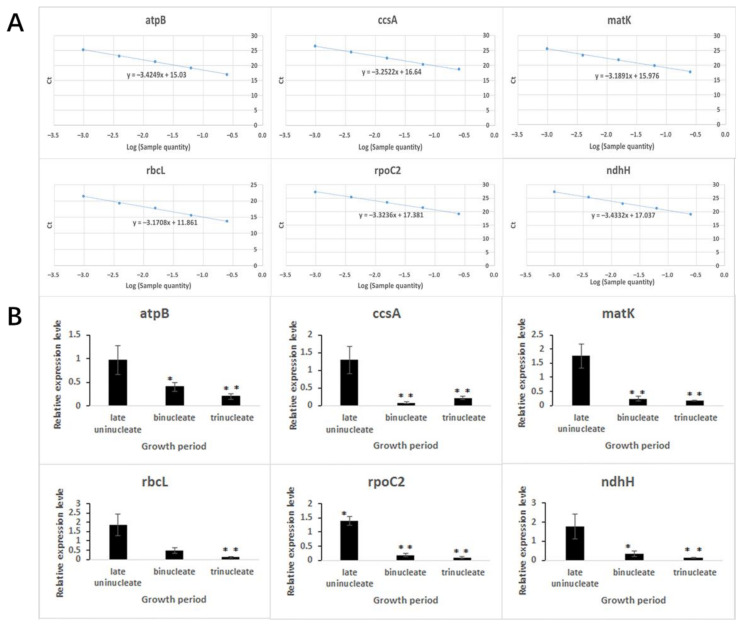
qPCR analysis for selected non synonymous mutant genes between K519A and 519B. (**A**) Standard curves of qPCR primers. The abscissa represents the Log (sample quantity). The ordinate represents the Ct values of primers. (**B**) Relative expression levels of selected non synonymous mutant genes in K519A compared with 519B. The abscissa represents the development period. The ordinate represents the expression level of related genes.

To further study the effect of nonsynonymous mutant genes in chloroplasts, we performed a qPCR analysis to assess the expression patterns of genes. According to the existing research results, we selected six nonsynonymous mutant genes (*atpB*, *ccsA*, *matK*, *rbcL*, *rpoC2*, and *ndhH*) for analysis of gene expression levels of K519A compared with 519B. The corresponding primers of qPCR are listed in Table 9. The efficiency of the qPCR primers calculated based on standard curve is 95.56–106.72% (Figure 7A).

It can be seen from the figures that the expression levels of all genes were downregulated at the binucleate and trinucleate stages, except that the *rbcL* gene was downregulated only at the trinucleate stage. Additionally, the *rpoC2* gene was upregulated at the late uninucleate stage (Figure 7B).

### 3.9. Function Analysis of Candidate Genes by BSMV-VIGS Method

**Figure 8:**
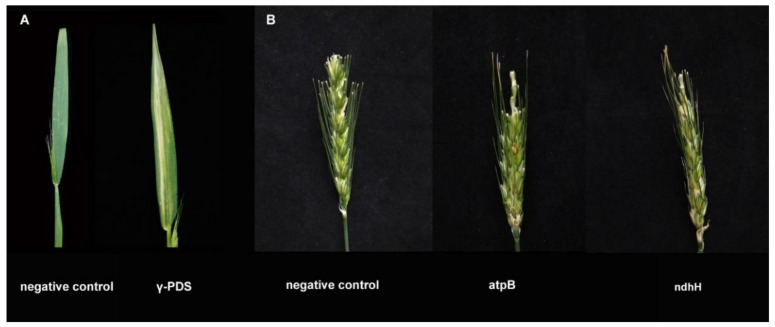
Phenotypic identification of gene silenced plants. (**A**) γ-PDS and negative control. (**B**) γ-gene and negative control.

To further study the relationship between chloroplast genes and fertility, several differentially expressed nonsynonymous mutant genes were selected for barley stripe mosaic virus-induced gene silencing (BSMV-VIGS) in 519B. First, the primers used for gene silenced were designed (Table 10). The recombinant vector was then successfully constructed. Albinism occurred in the leaves after *PDS*-silencing (positive control) (Figure 8A). It was concluded that the seed setting rate of *atpB*-silenced plants (20.7%) was significantly reduced compared with the negative control (93.8%). Additionally, the seed setting rate of *ndhH*-silenced plants (24.5%) was significantly reduced compared with the negative control (93.8%) (Figure 8B) (Table 11). This indicates that *atpB* and *ndhH* were likely to participate in the fertility transformation of 519B.

**Figure 9:**
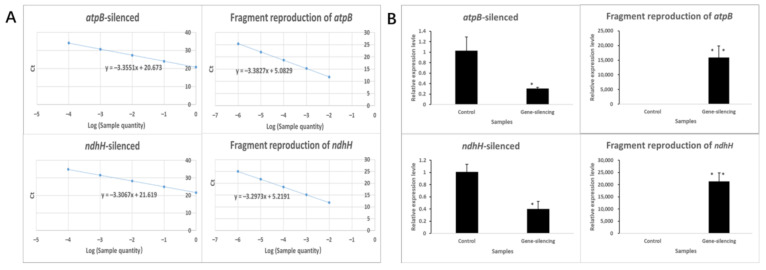
qPCR analysis of gene-silenced plants. (**A**) Standard curves of qPCR primers. The abscissa represents the Log (sample quantity). The ordinate represents the Ct values of primers. (**B**) Relative expression of gene-silenced plants. Values were calculated by the 2−^△^^CT^^△^^CT^ method, with ten biological duplicates and three technical duplicates and the bar represents SE.

Several spikelets at the binucleate stage were saved for qPCR. The primers for qPCR were listed in Table 10. Compared with the negative control individuals, the expression of *atpB* in the gene-silenced individuals was significantly reduced with atpB-qPCR primer, and the expression level was 0.31. After analysis of the reproduction of *atpB*-silenced fragment with atpB-VIGS primer, it was found that the reproduction efficiency was above 15,914. Compared with the negative control individuals, the expression of *ndhH* in the gene-silenced individuals was significantly reduced with ndhH-qPCR primer, and the expression level was 0.40. After analysis of the reproduction of *ndhH*-silenced fragment with ndhH-VIGS primer, it was found that the reproduction efficiency was above 21,283 (Figure 9).

## 4. Discussion

### 4.1. Plant Chloroplast Genomics Research and Phylogenetic Analysis

The chloroplast genome sequencing technology has developed from the FGS to the current third-generation sequencing [58,59,60]. Currently, most of the chloroplast genome sequences on NCBI are performed by second-generation sequencing technology [61,62]. Due to the fast improvement of sequencing technology, this results in sequencing costs continually decreasing, thus chloroplast genome data is largely supplemented. In the past few decades, chloroplast studies have made a breakthrough. More than 2400 plant chloroplast genomes have been published in the NCBI database. Of which, the chloroplast genome of tobacco is the first genome to be sequenced in higher plants [59]. The chloroplast genome encodes 100–120 genes, including 70–88 protein structural genes, 30–32 tRNA genes, and 4 rRNA genes. According to the functional classification of chloroplast genes, they can be divided into three categories: the first are genes encoding photoreactive structural proteins, such as *psaA*, *psbB*, *petD*, etc. The second are the coding genes of energy metabolism-related enzymes, such as *atpA*, *ndhB*, *rbcL*, and other genes. The third are the encoding genes of the transcriptional translation system, mainly including the ribosome large subunit encoding RPL family, the ribosome small subunit encoding RPS family, the transport RNA encoding TRN family, and transport RNA polymerase encoding RPOA family, and so on. Besides, there are some genes whose functions have not yet been determined, such as the YCF family [63]. Researches have shown that many genes of plant chloroplasts are involved in some important biological processes. For instance, Zhong et al. [64] demonstrated that the chloroplast heat shock protein HSP21 is involved in plastid-encoded RNA polymerase (PEP)-dependent transcription in *Arabidopsis*. Yu et al. [65] found that the downregulated expression of ribosomal protein S1 (RPS1) in *rps1* mutants negatively modulates the expression of heat-responsive gene *HsfA2* and its target genes in *Arabidopsis.*

Our study completed the sequencing of the chloroplast genome of the K-type CMS K519A and maintainer 519B. This is similar to the chloroplast genome structure of most plants, which is a typical four-segment structure. The chloroplast genome of angiosperms belongs to maternal inheritance and is generally not affected by genetic recombination. The chloroplast genome is much smaller than the nuclear genome and there are more copies in normal cells, so the chloroplast genome is easy to obtain. Additionally, the structure and coding genes of the chloroplast genome are relatively conserved [66,67].

### 4.2. SSR Molecular Marker

Molecular marker technology is to detect the polymorphism of DNA sequences based on PCR, which can directly compare the mutations of DNA sequences. With the continuous improvement of molecular biology technologies, it is becoming increasing mature and is applied in many research directions [68,69]. Among the molecular markers, the SSR marker has high variability, codominant inheritance, easy operation, and easy analysis [70]. It is widely used in genetic mapping construction, gene mapping, genetic diversity analysis, and variety identification of various crops. Stachel et al. [3] successfully analyzed the genetic diversity of 60 wheat cultivated varieties originating from three agro-ecological zones using microsatellite markers. Salameh et al. [71] used a molecular marker to transfer the resistant QTL (*Fhb1*) on the 5A chromosome into nine European winter wheat lines to develop new scab-resistant lines. Besides, Shim et al. [72] sequenced the whole chloroplast genome of millet and compared it with other reported chloroplast genomes, and obtained 125 SSR loci and 34 indel changes, which can provide effective information for the phylogeny. In our study, there were 186 possible SSR repeat motifs in K519A and 188 possible SSR repeat motifs in 519B. The accuracy of SSR markers and the probability of polymorphism among individuals in a population or among species can only be known when these SSR markers are used for genotyping. Therefore, we will collect some experimental materials for analysis.

### 4.3. Chloroplast Genes Related to Cytoplasmic Male Sterility

Some nuclear male sterility genes have been identified. For example, Xing et al. [21] cloned the *TaAPT2* gene related to thermo-sensitive sterility in wheat, and the gene expression levels of the young anthers, which during the fertility conversion stage, were significantly reduced under the sterile condition by Northern analysis, indicating that the *TaAPT2* gene may be related to fertility. Xia et al. [73] confirmed that the male sterile *ms2* mutant in wheat was caused by an insertion of terminal-repeat retrotransposons in a miniature (TRIM) element in the promoter region of the *Ms2* gene. In addition to this, some studies have shown that the deletion, duplication, or mutation of plant chloroplast DNA may cause the wrong transmission of genetic information between chloroplast, mitochondria and nucleus, leading to male sterility [74,75].

Some studies have found that some chloroplast genes in plants may affect the fertility of plants. For example, the *matK* gene is located in the intron of the chloroplast *trnK* gene. Jia et al. [76] extracted DNA of abortive flower buds from the radish male sterile line BT-18 and normal flower buds using DD-PCR technology to study the differential expression of RNA between abortive and normal buds in radish. Additionally, it was found that the *matK* gene appeared three times in different primers of PCR amplification, and the amplification bands were enhanced in abortive buds, which showed upregulated expression, indicating that the abortion of radish buds had a great relationship with *matK* gene. Ou et al. [77] studied the intron sequences of ORFs and *rps16* genes in chloroplasts of different CMS lines in rice. Additionally, they found that in the gametophyte sterility line, the *rps16* gene intron contains a GMTGAG sequence and a unique G at position 595, which can be used as a molecular marker to distinguish sporophytic sterility and gametophyte sterility. In this study, it was found that some genes had nonsynonymous mutant sites between K519A and 519B, and these genes might be related to male sterility.

The RNA polymerase is a key enzyme controlling chloroplast genomic transcription and gene expression. It is encoded by the copy genes *rpoA*, *rpoB*, *rpoC1,* and *rpoC2* genes [78]. The α subunit and β subunit of RNA polymerase are encoded by *rpoA* and *rpoB* genes, respectively, and the β’ and β” subunit by *rpoC1* and *rpoC2* genes. Chen et al. [79] found a new chloroplast DNA deletion in the sorghum CMS line, which occurs in the middle of the *rpoC2* gene, which may be associated with cytoplasmic male sterility in sorghum. We infer that nonsynonymous mutations of *rpoB* and *rpoC2* genes may affect the production of β and β” subunits of RNA polymerase.

### 4.4. Phylogenetic Relationship of Important Crops Based on Chloroplast Sequences

In recent years, with the continuous completion of plant chloroplast genome sequencing, phylogenetic researches based on genes or chloroplast genomes have been greatly promoted. Kallersjo et al. [80] utilized the *rbcL* chloroplast gene to analyze the phylogenetic relationship of 2538 species, covering the prokaryotic cyanobacteria to higher flowering plants, using the parsimony jackknife analysis method. Bruneau et al. [81] analyzed the evolutionary relationship of 223 legume materials by analyzing the introns of the chloroplast *trnl* gene. Kim [82] sequenced the chloroplast genome of *Panax schinseng* Nees and analyzed the evolutionary relationship of vascular plants. Liu [83] analyzed and reported the chloroplast genome of *Morella rubra* and the evolution patterns of Fagales, proving that Myricaceae is a sister to Juglandaceae. By comparing the chloroplast genomes of rice, wheat, and maize, it was found that some hot parts of the genes were susceptible to mutation, and the tandem tRNA region was species-specific. Additionally, gene deletion of the IR region and the boundary characteristics of the IR region indicated that the genetic relationship between rice and wheat is closer than that of maize [84]. Matsuoka et al. [85] studied 106 genes in the chloroplast genome of rice, wheat, and maize, and found that 86.8% of the genes showed the same evolution rate.

In this study, it was found that the chloroplasts of 101 materials from 13 genera were closely related. From the perspective of evolutionary relationship, *Hordeum* was the first to evolve, followed by *Eremopyrum*, *Henrardia*, *Australopyrum*, *Agropyron*, *Elymus*, *Thinopyrum*, *Dasypyrum*, *Secale*, *Aegilops speltoides*, *Triticum*, *Taeniatherum*, *Aegilops*, and *Amblyopyrum*. Middleton et al. [69] studied the phylogenetic relationships of the chloroplast genomes of 12 species and found that *Hordeum vulgare* divided into *Secale cereale* and wheat approximately 8–9 million years ago. It was consistent with this study. Saarela et al. [86] carried out phylogeny analysis on the chloroplast genomes of Triticinae and found that the differentiation relationship was in the order of *Hordeum jubatum, H. vulgare* subsp. *Vulgare, Connorochloa tenuis, Secale cereal, Taeniatherum caput-medusae, Ae. speltoides* var. *speltoides, T. timopheevii, T. aestivum, Triticum turgidum, Triticum macha, Triticum monococcum, Triticum urartu, Aegilops tauschii, Aegilops cylindrica, Aegilops geniculate, Aegilops longissima, Aegilops sharonensis, Aegilops bicornis, Ae. kotschyi,* and *Aegilops searsii.* This is almost consistent with the evolutionary relationships we found in the 13 genera. In addition, the *Triticum* contained hexaploid wheat and four origin species of hexaploid wheat in this study. Additionally, the order of differentiation is *T. timopheevii*, *T. Turgidum*, *T. urartu* and *T. monococcum*. In comparison with these four genera, the differentiation of *T. aestivum* is not successively obvious and almost simultaneous. The evolutionary sequence of *T. urartu* and *T. monococcum* was just opposite to the results of Saarela et al. [86], which may be due to the close relationship between the two species and the small differences in chloroplast genomes. In addition, previous studies found that the genome donors of hexaploid wheat diverged 2.1–2.9 million years ago [69]. It is not difficult to see from the successful application of next-generation sequencing technology in the acquisition of chloroplast genomic data in recent years that the emergence of this new technology will greatly promote the development of chloroplast phylogenetic analysis, and the phylogenetic research of plants will have a bright prospect [87,88].

## 5. Conclusions

In this study, we obtained and analyzed the three chloroplast genomes of two male sterile lines and one maintainer line in wheat. Based on a comparative analysis, a total of 104 mutations were found in 42 genes. There were 16 genes with nonsynonymous mutations between K519A and 519B. There were no base mutations in the protein encoding genes between K519A and YS3038. The *atpB*, *ccsA*, *matK*, *rbcL*, *rpoC2*, and *ndhH* genes were mostly downregulated at the binucleate and trinucleate stages. *AtpB* and *ndhH* were likely to be involved in the reproductive transformation of 519B. The chloroplast genomes of 519B and K519A were closely related to the *Triticum* genus and *Aegilops* genus, respectively.

## Figures and Tables

**Table 1:** Gene contents of K519A, YS3038, and 519B chloroplast genomes.

Category for Genes	Group of Genes	Gene Names
Genes for photosynthesis	ATP synthase	*atpA*, *atpB*, *atpE*, *atpF* *, *atpH*, *atpI*
	Cytochrome b/f complex	*petA*, *petB* *, *petD* *, *petG*, *petL*, *petN*
	NADH dehydrogenase	*ndhA* *, *ndhB* *, *ndhC*, *ndhD*, *ndhE*, *ndhF, ndhG*, *ndhH*, *ndhI*, *ndhJ*, *ndhK*
	Photosystem I	*psaA*, *psaB*, *psaC*, *psaI*, *psaJ*
	Photosystem II	*psbA*, *psbB*, *psbC*, *psbD*, *psbE*, *psbF*, *psbH*, *psbI*, *psbJ*, *psbK*, *psbL*, *psbM*, *psbN*, *psbT*, *psbZ*
	Large subunit of rubisco	*rbcL*
	ATP-dependent protease subunits P gene	*Clp P* **
Expression related gene	Proteins of small ribosomal (SSU)	*rps2*, *rps3*, *rps4*, *rps7*, *rps8*, *rps11*, *rps12* #, *rps14*, *rps15*, *rps16*, *rps18*, *rps19*
	Proteins of large ribosomal (LSU)	*rpl2* *, *rpl14*, *rpl16*, *rpl20*, *rpl23*, *rpl32*, *rpl33*, *rpl36*
	Ribosomal RNAs	*rrn4.5*, *rrn5*, *rrn16*, *rrn23*
	RNA polymerase	*rpoA*, *rpoB*, *rpoC1*, *rpoC2*
	Transfer RNAs	*trnA-UGC* *, *trnC-GCA*, *trnD-GUC*, *trnE-UUC*, *trnF-GAA*, *trnfM-CAU*, *trnG-GCC*, *trnG-UCC* *, *trnH-GUG*, *trnI-CAU*, *trnI-GAU* *, *trnK-UUU* *, *trnL-CAA*, *trnL-UAA* *, *trnL-UAG*, *trnM-CAU*, *trnN-GUU*, *trnP-UGG*, *trnQ-UUG*, *trnR-UCU*, *trnR-ACG*, *trnS-GCU*, *trnS-GGA*, *trnS-UGA*, *trnT-GGU*, *trnT-UGU*, *trnV-GAC*, *trnV-UAC* *, *trnW-CCA*, *trnY-GUA*
Other genes	Maturase	*matK*
	Envelope membrane protein	*cemA*
	C-type cytochrome synthesis gene	*ccsA*
	Translation initiation factor	*infA*
Proteins of unknown function	Conserved open reading frame	*ycf1*, *ycf2*, *ycf3* **, *ycf4*

*—Genes containing an intron. **—Genes containing two introns. #—Trans splicing genes.

**Table 2:** Codon preference analysis of K519A chloroplast genes.

Amino Acid Name	Total Number of Amino Acids	Total Number of Amino Acids
Ala(A)	832	GCU (37.26%), GCC (21.63%), GCA (25.96%), GCG (15.14%)
Arg(R)	1250	CGU (16.88%), CGC (10.08%), CGA (14.16%), CGG (7.84%), AGA (31.68%), AGG (19.36%)
Asn(N)	1043	AAU (70.85%), AAC (29.15%)
Asp(D)	595	GAU (74.29%), GAC (25.71%)
Cys(C)	468	UGU (53.21%), UGC (46.79%)
Gln(Q)	603	CAA (77.78%), CAG (22.22%)
Glu(E)	862	GAA (73.20%), GAG (26.80%)
Gly(G)	1166	GGU (29.07%), GGC (14.84%), GGA (35.59%), GGG (20.50%)
His(H)	562	CAU (78.83%), CAC (21.17%)
Ile(I)	1697	AUU (42.78%), AUC (25.34%), AUA (31.88%)
Leu(L)	1651	UUA (22.71%), UUG (26.83%), CUU (21.93%), CUC (10.60%), CUA (10.30%), CUG (7.63%)
Lys(K)	1148	AAA (66.90%), AAG (33.10%)
Met(M)	378	AUG (100.00%)
Phe(F)	1259	UUU (57.11%), UUC (42.89%)
Pro(p)	838	CCU (28.28%), CCC (24.70%), CCA (36.28%), CCG (10.74%)
Ser(S)	1866	UCU (21.01%), UCC (23.26%), UCA (6.91%), UCG (10.77%), AGU (19.13%), AGC (18.92%)
Thr(T)	1178	ACU (34.38%), ACC (28.61%), ACA (19.44%), ACG (17.57%)
Trp(W)	271	UGG (100.00%)
Tyr(Y)	851	UAU (64.98%), UAC (35.02%)
Val(V)	942	GUU (35.35%), GUC (15.61%), GUA (33.01%), GUG (16.03%)
Stop Codon	709	UAG (22.99%), UGA (18.62%), UAA (58.39%)

**Table 3:** Codon preference analysis of 519B chloroplast genes.

Amino Acid Name	Total Number of Amino Acids	Total Number of Amino Acids
Ala(A)	837	GCU (37.40%), GCC (21.74%), GCA (26.16%), GCG (14.70%)
Arg(R)	1250	CGU (16.80%), CGC (10.16%), CGA (14.56%), CGG (8.08%), AGA (31.04%), AGG (19.36%)
Asn(N)	1033	AAU (70.86%), AAC (29.14%)
Asp(D)	595	GAU (74.29%), GAC (25.71%)
Cys(C)	471	UGU (53.08%), UGC (46.92%)
Gln(Q)	601	CAA (77.54%), CAG (22.46%)
Glu(E)	861	GAA (73.17%), GAG (26.83%)
Gly(G)	1158	GGU (28.67%), GGC (14.94%), GGA (35.84%), GGG (20.55%)
His(H)	560	CAU (78.57%), CAC (21.43%)
Ile(I)	1692	AUU (42.43%), AUC (25.24%), AUA (32.33%)
Leu(L)	1675	UUA (23.28%), UUG (26.63%), CUU (21.61%) CUC (10.69%), CUA (10.15%), CUG (7.64%)
Lys(K)	1153	AAA (67.13%), AAG (32.87%)
Met(M)	380	AUG (100.00%)
Phe(F)	1261	UUU (56.78%), UUC (43.22%)
Pro(p)	848	CCU (28.42%), CCC (24.17%), CCA (37.62%), CCG (9.79%)
Ser(S)	1860	UCU (21.08%), UCC (23.33%), UCA (7.10%), UCG (10.54%), AGU (19.25%), AGC (18.71%)
Thr(T)	1177	ACU (34.41%), ACC (28.21%), ACA (19.46%), ACG (17.93%)
Trp(W)	268	UGG (100.00%)
Tyr(Y)	843	UAU (65.36%), UAC (34.64%)
Val(V)	948	GUU (35.55%), GUC (15.30%), GUA (33.44%), GUG (15.72%)
Stop Codon	710	UAG (23.80%), UGA (18.73%), UAA (57.46%)

**Table 4:** Characterization of simple sequence repeats discovered in the K519A chloroplast genome.

Microsatellite Sequences	Number of Base Repeats (SSR Number)	Microsatellite Sequences	Number of Base Repeats (SSR Number)
A	8(37), 9(10), 10(5), 11(3), 12(2), 13(3), 16(1), 23(1)	GCA	3(1)
C	8(2), 9(2)	GTT	3(2)
G	8(2), 9(1)	TAA	3(1)
T	8(25), 9(18), 10(9), 11(5), 13(1)	TAT	3(2), 4(1)
AT	5(3)	TCT	3(1)
TA	5(3)	TGC	3(1)
TC	5(2)	TTC	3(7), 4(1)
AAC	3(7)	TTG	3(1)
AAG	3(3)	AACG	3(1)
AAT	3(1), 5(1)	AAGA	3(1)
AGA	3(5)	AATA	3(1)
AGC	3(1)	AGAA	3(1)
AGT	3(1)	TCCT	3(1)
ATA	3(1)	TCGT	3(1)
CTT	3(1)	TTCA	3(1)
GAA	3(2)	TTCT	3(1)
GAT	3(1)	CCATA	3(1)

**Table 5:** Characterization of simple sequence repeats discovered in the 519B chloroplast genome.

Microsatellite Sequences	Number of Base Repeats (SSR Number)	Microsatellite Sequences	Number of Base Repeats (SSR Number)
A	8(40), 9(13), 10(3), 11(3), 12(3), 17(1), 18(1)	GTT	3(2)
C	8(3), 10(1)	TAA	3(1)
G	8(1), 10(1)	TAT	3(2), 4(1)
T	8(27), 9(13), 10(11), 11(2), 12(2), 13(1)	TCT	3(1)
AT	5(3), 6(1)	TGC	3(1)
TA	5(3)	TTC	3(6), 4(1)
TC	5(2)	TTG	3(1)
AAC	3(7)	AACG	3(1)
AAG	3(3)	AAGA	3(1)
AAT	3(1), 5(1)	AATA	3(1)
AGA	3(5)	AGAA	3(1)
AGC	3(1)	TCCT	3(1)
AGT	3(1)	TCGT	3(1)
ATA	3(1)	TTCA	3(1)
ATT	3(1)	TTCT	3(1)
CTT	3(1)	ATAGA	3(1)
GAA	3(2)	CCATA	3(1)
GAT	3(1)	TTTAT	3(1)
GCA	3(1)		

**Table 6:** Features and positions of long repeat fragments in the K519A and 519B.

K519A				519B			
Repeat Length	Starting Position	Match Direction	Starting Position	Repeat Length	Starting Position	Match Direction	Starting Position
286	56,620	F	134,618	286	56,605	F	133,861
77	0	F	136,919	263 *	56,628	F	133,884
40	66,331	F	66,373	77	0	F	136,158
38	38,456	F	40,680	40	65,528	F	65,570
30	87,709	F	130,393	38	38,432	F	40,656
30	130,393	P	130,393	30	86,907	F	129,632
36	43,404	F	90,709	30	129,632	P	129,632
36	43,404	P	127,387	36	43,403	F	89,907
33	66,338	F	66,380	36	43,403	P	126,626
29	7614	P	44,922	33	65,535	F	65,577
35	76,836	F	76,854	29	7613	P	44,931
35	76,845	F	76,863	35	76,031	F	76,049
37	66,351	F	66,393	35	76,040	F	76,058
33	27,173	F	27,224	37	65,548	F	65,590
29	27,177	F	27,228	33	27,181	F	27,232
34	27,251	F	27,272	29	27,185	F	27,236
34	66,373	F	66,394	34	27,259	F	27,280
25	80,343	F	80,388	34 *	27,321	F	27,396
31	27,376	F	27,442	34	65,570	F	65,591
31	66,389	F	66,410	25	79,542	F	79,587
33	27,256	F	27,277	31	27,384	F	27,450
30	12,940	F	36,397	31	65,586	F	65,607
30	16,817	P	16,817	33	27,264	F	27,285
30	66,787	P	66,787	30	12,893	F	36,373
32	27,132	F	27,231	30	16,815	P	16,815
32	27,309	F	27,384	30	65,984	P	65,984
32	27,313	F	27,388	32	27,140	F	27,239
29	11,348	P	44,925	32	27,287	F	27,341
26	27,138	F	27,237	32	27,317	F	27,392
31 *	27,114	F	27,384	29	11,303	P	44,934
31	27,301	F	27,442	26	27,146	F	27,245
28	36,250	R	36,250	31	27,309	F	27,450
22	16,821	P	16,821	28 *	27,327	F	27,402
25	7615	F	11,352	28	36,225	R	36,225
25	11,352	P	44,925	22	16,819	P	16,819
30	66,320	F	66,425	22 *	78,893	R	78,893
30	66,408	F	66,429	25	7614	F	11,307
27	27,160	F	27,355	25	11,307	P	44,934
27	66,373	F	66,415	30	65,517	F	65,622
21	7619	F	11,356	30	65,605	F	65,626
21	11,356	P	44,925	27	27,168	F	27,363
21	14,855	P	46,177	27	65,570	F	65,612
21	27,124	F	27,319	21	7618	F	11,311
21	113,039	R	113,039	21	11,311	P	44,934
29	27,092	F	27,146	21	14,864	P	46,185
29	38,447	F	40,671	21	27,132	F	27,327
26 *	27,319	F	27,394	21	112,256	R	112,256
26	77,132	F	77,150	24 *	27,259	F	27,334
26 *	107,460	P	107,460	29	27,100	F	27,154
20	7684	F	11,421	29	38,423	F	40,647
				26	76,327	F	76,345
				20	7683	F	11,376

F—forward. R—reverse. P—palindromic. C—complementary. * —Specific sequence for each line.

**Table 7:** Alignment analysis of coding genes of CS, K519A, YS3038, and 519B.

Genes	CS (Reference Sequence)	519B	K519A	YS3038	Genes	CS (Reference Sequence)	519B	K519A	YS3038
*atpA*	792C, 1164T	C, T	T, G	T, G	*psaB*	456G, 750C, 940C, 1155G, 1644G	G, C, C, G, G	A, G, A, C, A	A, G, A, C, A
*atpB*	35C-12A, 56G-19S, 164A-55D, 321T, 651G, 1215T, 1489C-497Q	C-A, A-N, A-D, T, G, T, C-Q	T-V, G-S, C-A, C, A, C, A-K	T-V, G-S, C-A, C, A, C, A-K	*psbB*	1188C, 1523C-508A	C, C-A	T, T-V	T, T-V
*atpE*	54G	G	A	A	*psbC*	147A	A	G	G
*atpF*	378C	C, 146D:15	T, 146D:15	T, 146D:15	*psbD*	558T, 738A	C, A	C, C	C, C
*atpI*	225C, 477C-159S, 555A	C, A-R, A	T, C-S, G	T, C-S, G	*psbH*	133G-45V	G-V	A-I	A-I
*ccsA*	78G-26L, 553C-185L, 858C	G-L, C-L, T	T-F, T-F, T	T-F, T-F, T	*psbI*		3D:36	3D:36	3D:36
*cemA*	642C	C	T	T	*psbJ*	51C	C	T	T
*clpP*	210C, 309A	G, A	A, G	A, G	*psbZ*	126T	T	C	C
*infA*	none	4I:93	4I:93	4I:93	*rbcL*	40A-14K, 284C-95S, 1429A, 1431G	A-K, G-S, A, G	C-Q, A-N, T, A, 1432I:3	C-Q, A-N, T, A, 1432I:3
*matK*	54C, 100G-34D, 423T-141F, 537G, 564C, 608T-203F, 927A-309L, 1377G, 1465T-489C	C, G-D, T-F, A, C, T-F, C-F, A, T-C	T, C-H, A-L, G, T, A-Y, C-F, A, A-S	T, C-H, A-L, G, T, A-Y, C-F, A, A-S	*rpl14*	326G-109G	G-G	A-E	A-E
*ndhA*	44G-15W, 102C, 444G, 550T	G-W, T, G, C	C-S, C, A, C	C-S, C, A, C	*rpl16*	4T, 5A, 8A, 9C, 237A	C, T, G, T, A, 4D:81	C, T, G, T, G, 4D:81	C, T, G, T, G, 4D:81
*ndhB*	783T	T	C	C	*rpl2*	none	1I:336	1I:336	1I:336
*ndhC*	84A, 276C	A, C	T, T	T, T	*rpl32*	172A-58N, 186T-62F	A-N, T-F	G-D, G-L	G-D, G-L
*ndhD*	240G	A	G	G	*rpoA*	219A, 625C	A, C	G, T	G, T
*ndhE*	264G	G	A	A	*rpoB*	438G, 459A, 1245A, 1284C, 1366G-456E, 1899T, 1908A, 2628A, 2937G	G, A, A, T, G-E, T, A, A, G	C, G, G, T, A-K, C, G, G, C	C, G, G, T, A-K, C, G, G, C
*ndhF*	661G-221V, 768A, 783A, 1764T, 1989T, 2052A, 2127A	G-V, A, G, T, T, A, A	A-I, G, G, C, C, C, G	A-I, G, G, C, C, C, G	*rpoC1*	712C, 783A, 1036A, 1683T	C, A, A, T	A, T, C, C	A, T, C, C
*ndhH*	519G-173E, 793G-265V	G-E, G-V	T-D, A-I	T-D, A-I	*rpoC2*	351C, 1002A, 1695A, 2083C-695P, 2125A-709I, 2223T, 2780C-927S, 2984A-995D, 3564C, 3789G, 4074G, 4334T-1445I, 4413C	C, A, T, C-P, A-I, T, C-S, A-D, C, A, G, T-I, C	A, G, A, T-S, C-L, C, T-F, G-G, T, G, A, A-K, T	A, G, A, T-S, C-L, C, T-F, G-G, T, G, A, A-K, T
*ndhH-2*	none	211	211D:6	211D:6	*rps14*	132T, 276A	G, A	G, G	G, G
*ndhI*	232C	C	T	T	*rps16*	none	120G, 177C-59S, 178A-60T, 4D:81	A, A-R, G-A, 4D:81	A, A-R, G-A, 4D:81
*ndhJ*	54A	A	G	G	*rps18*	162T	T	C	C
*ndhK*	96T, 484G-162G	T, G-V, 4D:60	C, A-I, 4D:60	C, A-I, 4D:60	*rps2*	552G	A	G	G
*petA*	606C, 666A	T, A	C, G	C, G	*rps3*	90G, 597C	G, C	T, T	T, T
*petB*	5A, 252C	G, C, 5D:51	G, A, 5D:51	G, A, 5D:51	*rps4*	397A	A	C	C
*petD*	4C, 5C, 7A, 297C, 360T	G, G, G, C, T, 4D:42	G, G, G, T, C, 4D:42	G, G, G, T, C, 4D:42	*rps8*	108G	T	T	T

146D:15 means that this line is deleting 15 bases starting at the 146th base position, and the following is the same. 4I:93 means that this line is inserting 93 bases starting at the fourth base position, and the following is the same. 35C-12A means that the 35th base in this line is C, which corresponds to the 12th amino acid, A, and the following is the same.

**Table 8:** Primers for first-generation sequencing.

Genes	Forward Primers	Reverse Primers
*matK*	TCAACCCTTTCCTGTTTCTT	CGTCAACAATACTTTGTCTACC
*rps16*	GCTTATGTTGGATTGGCACGAT	CCCGAAGTAATGTCTAAACCCA
*rpoB*	CGGATTGGCTCTTGGTCGTT	CGATTCGCATCATTGTGCTCAA
*rpoC2*	GGAATCCTAGAAGACGAATACG	GACCTGTTAGTGTTCTAAGTTC
*atpI*	TCCCTGGGTTCCCTTTATTGG	AGAGCTTGAATACCGCTTGTAA
*ndhK*	GCAATTCTTGCTCATAAGTTCC	GGGAATGTTCAGTACGGATTC
*atpB*	GGACCCATATCTACTGCTGTGT	GGGCAACGAAATCAAGTCCTG
*rbcL*	CATACACAGGGTGTACGCATTA	AGATTGAGCCGAGTTTAATTGC
*psbB*	TGGTGGCGAACTTAATGGAGTA	TTGGAGTTGGTGGAACCTTAGG
*psbH*	GGCGACTAAAGTTGCTGTTTCC	CAGTCTGGTTGCGAGGTCTTAA
*rpl14*	GGGTCGGCTGATACGCTTTA	ATGATGACAATGCTGCGGTTAT
*ndhH*	GCCAGCATATTCAGGACCAAT	AGAGCGAGTTGAAGGAGTAGG
*ndhF*	AGAGGAAGAAGTCGAGCTAGAA	TGGTCTTGGACCGTCAATAATG
*Rpl32*	GCAGCAATAGATGTCTTTCACA	TGGAGAAGATAGGTGGAAGTTG
*ccsA*	CGAGTGGCGGCATTCTTGA	TGGAAATGAAGGGACAGAGGTT
*ndhA*	GCTTCCGAATTGATCTCATCCT	AGTTAGTGAAGGGTTAGGAACA

**Table 9:** Primers for the qPCR.

Genes	Forward Primers	Reverse Primers
*atpB*	TTGATAACCCACTGCGGAGG	CTGCCCTAACTATGGCGGAA
*ccsA*	CGGAGCGTTTGGATTCTTGG	GCCTCATTAGCCCATACTGC
*matK*	AGCGCATGAAAGTCGAAGTA	TCTTGATCGATTTGGTCGGA
*ndhH*	GTAGATCGGCGGCTACTCCT	TCGGCGCACAGACTCCTTT
*rbcL*	TGAATGCGACTGCGGGTACA	AGGCCATTGTCGCGGCAATA
*rpoC2*	GGCTGGTGCTTCCCTTGTTG	ATCGGTCCTGCACTTGTCCT

**Table 10:** Primers for the BSMV-VIGS of genes and qPCR for silenced individuals.

Name	Forward Primers	Reverse Primers
atpB-VIGS	CCTTAATTAAGCAGGGTCGGTCAAATCGT	ATAAGAATGCGGCCGCTTGTTCAAGCAGGATCAGAGGT
ndhH-VIGS	CCTTAATTAAACCTACTCCTTCAACTCGCTCT	ATAAGAATGCGGCCGCGCTGCTACAGGTATGCGAATGA
atpB-qPCR	TTGATAACCCACTGCGGAGG	CTGCCCTAACTATGGCGGAA
ndhH-qPCR	GTAGATCGGCGGCTACTCCT	TCGGCGCACAGACTCCTTT

**Table 11:** Seed setting rate of silenced plants.

	*atpB*-Silenced Plants			*ndhH*-Silenced Plants			Negative Control Plants			
	Grain Number	Effective Spikelet Number	Seed Setting Percentage (%)	Grain Number	Effective Spikelet Number	Seed Setting Percentage (%)	Grain Number	Effective Spikelet Number	Seed Setting Percentage (%)	Statistical *P*
	6	10	30.0%	4	8	25.0%	17	10	85.0%	1.34448 × 10^−56^
	3	12	12.5%	5	9	27.8%	17	9	94.4%	
	6	10	30.0%	3	9	16.7%	18	10	90.0%	
	4	10	20.0%	7	10	35.0%	15	8	93.8%	
	3	8	18.8%	5	9	27.8%	18	10	90.0%	
	5	10	25.0%	4	10	20.0%	19	10	95.0%	
	3	9	16.7%	2	7	14.3%	16	8	100.0%	
	3	10	15.0%	6	10	30.0%	18	9	100.0%	
	4	10	20.0%	3	8	18.8%	17	9	94.4%	
	3	8	18.8%	6	10	30.0%	21	11	95.5%	
Average			20.7%			24.5%			93.8%	

## Data Availability

The sequences of mitochondrial genomes have been deposited in the GenBank database at National Center for Biotechnology Information (NCBI) (https://www.ncbi.nlm.nih.gov/genbank/, accessed on 30 November 2021) and can be accessed by the accession numbers MN605257, MN605258, and OL678073 for K519A, 519B, and YS3038, respectively.

## Abbreviations

BSMV-VIGS	Barley stripe mosaic virus-induced gene silencing
CMS	Cytoplasmic male sterility
GMS	Genic male sterility
FGS	First-generation sequencing
